# Wood-water relationships and their role for wood susceptibility to fungal decay

**DOI:** 10.1007/s00253-020-10479-1

**Published:** 2020-03-06

**Authors:** Christian Brischke, Gry Alfredsen

**Affiliations:** 1 Department of Wood Biology and Wood Products, Faculty of Forest Sciences and Forest Ecology, University of Goettingen, Buesgenweg 4, D-37077 Goettingen, Germany; 2 Norwegian Institute of Bioeconomy Research (NIBIO), Division of Forest and Forest Resources, Wood Technology, Høgskoleveien 8, 1433 Ås, Norway

**Keywords:** Brown rot, Durability, Fungi, Minimum moisture threshold, Physiological limit, Pile test, Soft rot, Sorption, White rot, Wood decomposition, Wood-decay fungi, Wood-water interactions

## Abstract

**Abstract:**

Wood in service is sequestering carbon, but it is principally prone to deterioration where different fungi metabolize wood, and carbon dioxide is released back to the atmosphere. A key prerequisite for fungal degradation of wood is the presence of moisture. Conversely, keeping wood dry is the most effective way to protect wood from wood degradation and for long-term binding of carbon. Wood is porous and hygroscopic; it can take up water in liquid and gaseous form, and water is released from wood through evaporation following a given water vapour pressure gradient. During the last decades, the perception of wood-water relationships changed significantly and so did the view on moisture-affected properties of wood. Among the latter is its susceptibility to fungal decay. This paper reviews findings related to wood-water relationships and their role for fungal wood decomposition. These are complex interrelationships not yet fully understood, and current knowledge gaps are therefore identified. Studies with chemically and thermally modified wood are included as examples of fungal wood substrates with altered moisture properties. Quantification and localization of capillary and cell wall water – especially in the over-hygroscopic range – is considered crucial for determining minimum moisture thresholds (*MMThr*) of wood-decay fungi. The limitations of the various methods and experimental set-ups to investigate wood-water relationships and their role for fungal decay are manifold. Hence, combining techniques from wood science, mycology, biotechnology and advanced analytics is expected to provide new insights and eventually a breakthrough in understanding the intricate balance between fungal decay and wood-water relations.

**Key points:**

• Susceptibility to wood-decay fungi is closely linked to their physiological needs.

• Content, state and distribution of moisture in wood are keys for fungal activity.

• Quantification and localization of capillary and cell wall water in wood is needed.

• New methodological approaches are expected to provide new insights

## Introduction

Wood is the largest pool of above-ground terrestrial carbon, and fungi dominate the recycling of this sequestered carbon (Zhang et al. 2019a, b). Wood-decaying fungi have traditionally been assigned to three major groups referring to the macroscopic and microscopic degradation pattern they form in wood, i.e. brown-rot, white-rot and soft-rot decay. However, based on genome comparison, Riley et al. (2014) found ‘a continuum rather than a dichotomy between the white-rot and brown-rot modes of wood decay’, where brown-rot fungi are a polyphyletic group evolved from at least seven white-rot lineages (Floudas et al. 2012; Hibbett and Donoghue 2001; Zhang et al. 2019c). But the terms brown-, white- and soft-rot are still widely used because they are providing information about general decay mechanisms.

Since brown-rot fungi have a lower repertoire of known enzymes than white-rot fungi (Riley et al. 2014), brown-rot fungi have historically been less studied than white-rot fungi. The details about brown-rot mechanisms are still under discussion, but it is generally agreed that brown-rot fungi use a two-step oxidative-enzymatic mechanism (Wei et al. 2010; Korripally et al. 2013; Arantes and Goodell 2014; Zhang et al. 2016), and efforts are now done to reveal more about the mechanisms (e.g. Goodell et al. 2017; Presley and Schilling 2017; Zhang and Schilling 2017; Castaño et al. 2018; Presley et al. 2018; Wu et al. 2018; 2019; Zhang et al. 2019a, b, c). Brown-rot fungi prefer conifers and degrade hemicellulose and cellulose while leaving a modified (brown) lignin-rich residue behind (Cowling 1961; Filley et al. 2002; Pandey and Pitman 2003; Arantes and Goodell 2014). Even if brown-rot fungi lack > 60% of the genes known to be involved in white rot, they degrade wood at a higher rate than white-rot fungi in monocultures in laboratory (Castaño et al. 2018). In general, brown-rot fungi have greater effects on the elastomechanical properties of wood than white-rot fungi (Winandy and Morell 1993).

Complete lignin degradation is mainly known for white-rot fungi, but there are also instances of brown-rot fungi degrading significant portions of lignin such as *Gloeophyllum trabeum* (Kaffenberger and Schilling 2013). Since lignin is a recalcitrant material, white-rot decay and the enzymes involved have been explored in more detail, lately with focus on biorefinery utilization. White-rot fungi prefer deciduous trees and use powerful oxidative and hydrolytic enzymes that gradually degrade cellulose while lignin is completely mineralized, leaving lighter coloured (white) cellulose behind (Riley et al. 2014). The major lignin-degrading enzyme systems of white-rot fungi include lignin peroxidase, manganese peroxidase, versatile peroxidase and laccase (Manavalan et al. 2015).

While brown and white rot is caused by basidiomycetes, soft-rot is caused by ascomycetes and fungi imperfecti. Soft-rot fungi primarily degrade hemicelluloses and cellulose; lignin degradation is less extensive than by white-rot fungi. The decomposition process can lead to formation of cavities inside the cell wall and sometimes a discoloration and cracking pattern like that of brown-rot fungi (Manavalan et al. 2015). At advanced stages of decay, the lignin-rich middle lamella is left behind as a skeleton of the wood structure with low strength properties (Daniel and Nilsson 1997; Kim and Singh 2000). Soft rot tends to occur in environments where basidiomycetes are restricted by factors such as low aeration, high moisture levels or high temperatures (Goodell et al. 2008; Manavalan et al. 2015).

Wood will also get attacked by blue stain and mould, but they are regarded mainly as an aesthetical issue since they primarily use the easily available nutrients (sugars) in the wood and do not degrade the structural biopolymers. Moulds only cause superficial discoloration while the dark-coloured hyphae of blue-stain fungi give dark discolouration of the sapwood. Blue-stain fungi degrade the pit membranes, and this causes increased water permeability. Beyond that, moulds can become an indoor problem with respect to allergic reactions of inhabitants against their spores (Crook and Burton 2010).

The degradation of the different wood constituents such as hemicelluloses, cellulose, and lignin are partly enzymatic, partly oxidative and still not entirely understood, but differ between decay types and partly also between fungal species. However, moisture has been recognized as a key parameter and governing factor for fungal growth and decomposition of wood. It has long been agreed that liquid water (i.e. capillary water) is a prerequisite to allow extracellular transport of fungal metabolites and subsequent breakdown of cell wall components.

Wood is porous and hygroscopic; it can take up water in liquid and gaseous form, and water is released from wood through evaporation following a given water vapour pressure gradient. Wood moisture content (*MC*) is not the mass fraction of water, but rather the ratio of water mass (total mass of water in the wood) to wood mass (the dry mass of the wood alone). Therefore, *MC* of over 100% is possible if the mass of the water is greater than the mass of the wood itself. *MC* and wood-water interactions affect several wood properties important for applied purposes like strength and stiffness properties (Tiemann 1906), dimensional stability (Stamm 1959), biological degradation (Schmidt 2006) and fastener corrosion (Zelinka and Rammer 2009; Jakes et al. 2013).

Different models exist to describe absorption and desorption processes as well as different moisture states when wood is in equilibrium with the ambient air. In contrast, wood in outdoor applications undergoes frequent changes of wetting and drying, and sorption of water in the vapour phase can be easily overruled by liquid water uptake due to precipitation or condensation. During the last three decades, the perception of wood-moisture-relationships changed significantly and so did the view on moisture-induced properties. Among the latter is the susceptibility of wood to fungal decay. Many new insights on the mode of fungal action and the effect of moisture during wood decay were derived from studies with chemically and thermally modified wood. This paper reviews the state of the art of wood-water relationships and their role for decomposition by wood-destroying fungi. These are complex interrelationships not yet fully understood, and current knowledge gaps are therefore identified.

## Physiological needs of wood-decay fungi

### Historic insight

The process of wood infestation by decay fungi can be divided into different phases. The authors suggest the following: (1) spore arrival, (2) spore germination, (3) mycelial growth, (4) wood metabolism, (5) autolysis of fungal hyphae and (6) formation of fruiting bodies and sporulation. It is assumed that the requirements regarding moisture and other physico-chemical parameters (e.g. pH, temperature, nutrients) differ between the six phases. But the six phases of fungal infestation will overlap in the wood substrate because of spatial colonization, and the required physico-chemical factors can also overlap between phases of wood decomposition. Most relevant for wood in service – especially in above-ground situations – and therefore in the focus of wood pathologists are the phases of spore germination, mycelial growth and metabolization of wooden cell walls. Wood exposed in soil contact is often in direct contact with fully developed fungal mycelium, and the phases of spore arrival and spore germination is only relevant for the transition zone between soil and air.

In the following, a chronological synopsis is given on methods, thresholds and experimental limitations regarding the moisture requirements for fungal growth and decay in wood based on a literature review by Brischke et al. (2018a). Several authors starting in the 1850s performed experiments where wood specimens were subjected to different climatic conditions, and spore germination or mycelial growth were monitored (Zeller 1920). Moisture requirements were often in the focus (e.g. Münch 1909; Wehmer 1914). Since then, thresholds for fungal growth and decay of wood were sought in numerous research works, where the experimental set-ups differed in external moisture supply and the way of infecting the wood specimens, and consequently, various minimum moisture thresholds (*MMThr*) were determined for different combinations of wood and fungal species.

Among the first, Zeller (1920) reported on the relationship between relative humidity (*RH)* and spore germination of wood-destroying fungi and found that the percentage of germinating spores of the brown-rot *Lenzites saepiaria* (syn. *Gloeophyllum sepiarium*) escalated above 90% *RH*, i.e. below the fibre saturation point (*FSP)*, which he considered to be at 95% *RH*. It is important to keep in mind in the following that the terminology regarding the *FSP* is not consistent in literature and refers to different moisture states. For more discussion see the ‘Cell wall saturation’ section below. Butin (1962) reported about germination experiments with spores of the ascomycete *Cryptodiaporthe populea* at varying vapour pressure, and the results aligned with the basidiomycete findings by Zeller (1920). Ascospores on malt agar germinated at 20 °C between 100 and 89% *RH* and conidia between 100 and 95.5% *RH*. However, the application of spores on wooden substrates at a given moisture content (*MC*) is challenging. Usually, for this purpose, spores are dispersed in water, and an aqueous spore suspension is sprayed or otherwise applied on the wood surface, which inevitably leads to a superficial increment in moisture. The latter can be reversed by rapid re-drying. However, it is also challenging to produce viable spores in sterile laboratory conditions. Alternatively, spores can be allowed to drop from fruiting bodies directly on wood samples as reported by Zeller (1920), but the method bears a high risk of contamination by non-target organisms such as mould fungi and bacteria.

It has been frequently shown that fungal spores were able to germinate at *RH* below 95% (Gottlieb 1950) corresponding to wood *MC* below fibre saturation. One might hypothesize that this also allows for the colonization of the wood substrate with fungal mycelium, but to the authors knowledge, evidence from experimental studies is still lacking. Other factors such as pH, oxygen content, volatile organic compounds and temperature are likely affecting both the germination of spores (Zeller 1920; Gottlieb 1950; Merrill 1970; Viitanen 1994) and the formation of mycelium, but their effects are not necessarily the same. Wood protection systems will obviously also alter the wood substrate by adding chemicals that are toxic for the fungi and/or by changing the wood-water properties.

### Minimum moisture thresholds (MMThr)

An extensive chronology of experimental studies to determine the moisture requirements for mycelial growth and wood decomposition by different wood-destroying basidiomycetes has been provided by Brischke et al. (2018a). A brief and updated summary is provided below. An overview of corresponding *MMThr* values is given in Table 1.

**Table 1:** Minimum moisture thresholds (*MMThr*) for fungal decay as determined in different studies using different test climates and set-ups with different ways of moisture supply

Inoculation of specimens	Moisture source	Parameters when *MMThr* at *ML* ≥ 2% was achieved						Reference
		*ML [%]*	*T [°C]*	*RH [%]*	Wood species	Fungus	*MC*_i_ [%]	
Sawdust, pre-inoculated with mycelium	Humidity, sawdust	2.2	20	85.6	*P. sylvestris* sW	*Coniophora cerebella*	–	Bavendamm and Reichelt (1938)
Sawdust, pre-inoculated with mycelium	Humidity, sawdust	2.2	20	85.6	*P. sylvestris* sW	*Coniophora cerebella*	–	Theden (1941)
Specimens, pre-inoculated with mycelium	Humidity, mycelium was allowed to grow into liquid	3.0	20	98.2	*P. sylvestris* sW	*Lenzites abietina*	28.0	Ammer (1963)
Specimens, pre-inoculated with mycelium	Humidity, increased *MC* of specimens due to pre-infection on malt agar	2.4	20	85.0	*Picea abies* sW	*Coniophora puteana*	19.0	Saito et al. (2012)
Specimens, pre-inoculated with mycelium	Humidity, increased *MC* of specimens due to pre-infection on malt agar	–	–	–	–	–	–	Brischke et al. (2017)
Piled specimens, mycelium	Contact to water or liquid malt agar, humidity	2.0	–	–	*P. sylvestris* sW	*Physisporinus vitreus*	31.0	Schmidt et al. (1996)
Piled specimens, mycelium on malt agar	Contact to malt agar, humidity	2.0	–	–	*P. sylvestris* sW	*Serpula lacrymans*	26.2	Huckfeldt et al. (2005)
Piled specimens, mycelium on malt agar	Contact to malt agar, humidity	5.4	–	–	*P. sylvestris* sW	*Coniophora puteana*	27.4	Stienen et al. (2014)
Piled specimens, mycelium on malt agar	Contact to malt agar, humidity	2.2	–	–	*F. sylvatica*	*Trametes versicolor*	15.4	Stienen et al. (2014)
Piled specimens, pre-inoculated specimens at bottom of pile	Presence of malt agar, humidity	2.5	–	–	*Q. robur* sW	*Serpula. lacrymans*;	25.5	Höpken (2015)
Piled specimens, pre-inoculated specimens at bottom of pile	Presence of water, humidity	2.9	–	–	*Q. robur* sW	*Serpula lacrymans*	24.9	Höpken (2015)
Piled specimens, pre-inoculated specimens at bottom of pile	Humidity	2.0	–	–	*Picea abies*	*Trametes versicolor*	16.3	Brischke et al. (2017)
Piled specimens, non-sterilized, non-inoculated	Presence of water, humidity	–	–	–	–	–	–	Vanpachtenbeke (2019)
Specimens, non-sterilized, non-inoculated	Humidity	0.0	25	9	*Picea abies,* *P. sylvestris* sW	–	–	Vanpachtenbeke (2019)
Specimens between two climates, non-sterilized, non-inoculated	Humidity, interstitial condensation	0.0	5 25	80 97	*Picea abies,* *P. sylvestris* sW	–	–	Vanpachtenbeke (2019)

*P.* *Pinus; F* *Fagus; Q* *Quercus*; *sW* sapwood; *ML*: mass loss; *RH* relative humidity; *MC*_*i*_ wood moisture content after incubation

Several experiments to determine *MMThr* for fungal growth and decay were performed using saturated salt solutions to establish well-defined climates and monocultures of wood-destroying fungi. Bavendamm and Reichelt (1938) conducted fungal growth tests on malt agar with wood saw dust and small wood blocks at different *RH* between 81.5 and 99% in small jars. Sodium chloride solutions of different concentration were used to obtain defined climates. Wood specimens were infested using pre-inoculated saw dust. After 4 months of exposure, more than 2% mass loss (*ML*) was detected on blocks stored at only 85.6% *RH*, but the *MC* after incubation was not determined. Theden (1941) determined the *MMThr* for new infection through mycelium, progress of decay in already incubated samples and reactivation of decay in infected, dried, and remoistened samples. The *MMThr* for onset of fungal decay was achieved at 98.2% *RH* for different test fungi. The higher the *ML* by fungal decay, the higher was the *MC* after incubation, which Theden (1941) explained by the production of water during the biochemical degradation of wood. In summary, Theden (1941) did not determine a *MMThr* below *fibre saturation*, even though decay started at *RH* below 100%. Similar discrepancies between target *MC* and actual *MC* after incubation were reported by Ammer (1963), who used pre-inoculated specimens and stored them in screw-top jars above different saturated salt solutions. Ammer (1963) examined Norway spruce (*Picea abies*) sapwood and determined at 85% *RH* an *MMThr* of 19% for fungal decay, which was approximately 7% points below its *FSP*. In a similar set-up, Saito et al. (2012) exposed specimens made from Japanese red pine (*Pinus densiflora*) in small vessels with even smaller containers filled with different saturated salt solutions. In contrast to the above-cited studies, no decay was observed at *MC* below fibre saturation.

In a different approach, a wide range of wood *MC* was generated by piling wood specimens in Erlenmeyer flasks where the bottom of the piles was exposed to malt agar inoculated with fungal mycelium serving as nutrition and water source at the same time (Schmidt et al. 1996; Huckfeldt et al. 2005; Huckfeldt and Schmidt 2006; Stienen et al. 2014; Meyer and Brischke 2015; Meyer et al. 2015b). The test fungus stopped growing upwards where moisture was insufficient, and *ML* decreased with the pile height. Within all the above-mentioned studies using the piling method, the *MMThr* were below *FSP* (Table 1), partly remarkably far below *FSP*. For instance, Meyer et al. (2015b) found a lower moisture limit for decay (*ML* = 2.2%) of beech wood by the white-rot *Trametes versicolor* of only 15.4% *MC*. However, the malt agar at the bottom of the pile served as an external moisture and nutrition source. The fungus is able to transport water and likely nutrition from the agar pile upwards through mycelium and strains, which can barely reflect the real-life situation for decay fungi on wood exposed above ground. In contrast, a permanent source of water and nutrients is available when wood is exposed in soil. Hence, Höpken (2015) modified the pile test method to examine the ability of decay fungi to transport water. Capillary water transport in the pile was interrupted by stainless steel washers between the wood specimens, and tests were conducted with and without malt agar. Höpken (2015) clearly showed that different fungi could actively transport water within the piles.

Brischke et al. (2017) determined *MMThr* in different experiments without an external moisture source. These tests referred to the experimental set-up suggested by Ammer (1963) using different saturated salt solutions and to the pile tests conducted by Meyer and Brischke (2015), but omitting malt agar as nutrition and moisture source. The *MMThr* for *T. versicolor* that caused significant *ML* on beech was achieved at 96% *RH*, i.e. at 25.3% *MC*, when specimens were conditioned above saturated salt solutions and deionized water, respectively, before inoculation with basidiomycete mycelium. Piled Norway spruce specimens showed significant *ML* already at 16.3% *MC* caused by *T. versicolor* without external supply of liquid water.

Vanpachtenbeke (2019) abstained from the use of any pre-infection with decay fungi and exposed wood specimens at given climates for several months. In so-called fungal control units (FCU), wood samples were exposed to high humidity (25 °C, 97% *RH*). In a second set-up, two modules (25 °C, 97% *RH* and 5 °C/80% *RH*) were separated by mineral wool and a wind barrier. The vapour pressure gradient between the modules allowed for interstitial condensation and thus moistening of the wood specimens. However, in both FCU, no fungal decay occurred during 3, 9, and 12 months of exposure, respectively.

Vanpachtenbeke (2019) also studied fungal decay in wood specimens with different initial *MC* at different *RH* compared to specimens incubated at 100% *RH*. The effect of *RH* on *ML* became evident, but it was also shown that within a few days even at low *RH* (e.g. 43%) the *MC* increased rapidly above *FSP* which can be attributed to active moisture transport from the malt agar by the brown-rot fungus *C. puteana*.

To determine the moisture requirements of wood and decay fungi is challenging. Besides the various limitations with fungal experiments and the difficulties to determine wood *MC* accurately, it appeared that the most challenging task is the interpretation of the test results. Rather often, the origin and the exact location of water in wood stay unclear. The latter is closely related to the relationship between air humidity and the equilibrium moisture content (*EMC*) of wood. However, different physico-chemical processes are involved in wetting and drying of wood. Hence, the moisture requirements of decay fungi cannot be reduced to static wood *MC* values but need to be seen in the context of dynamic processes including adsorption, diffusion, capillary condensation, desorption, and active moisture transport by the fungus itself. Usually, only an average wood *MC* (global *MC*) is measured, and *MC* gradients between different locations in wood (local *MC*) are barely accounted for (Meyer et al. 2015a). Finally, fungal degradation of wood itself supplies moisture.

Research often focussed on the question whether fungal decay can be initiated below fibre saturation or in other words whether capillary water in the cell lumens or other larger voids in the cell wall is needed for fungal decay. However, the definition of fibre saturation is somewhat diffuse and changed a lot during recent years and so did the understanding of wood-water relationships.

## Wood-water relationships

The interrelationship between wood and water has been subject to research for more than a century, and scientific literature on the topic was reviewed at irregular intervals (e. g. Venkateswaran 1970; Skaar 1988; Hartley et al. 1992; Engelund et al. 2013; Thybring et al. 2019). Established models and theories were critically and controversially discussed (Fredriksson and Thybring 2018, 2019; Zelinka et al. 2018) coming along with new methods and techniques for analysing sorption processes and localizing water inside wood tissues and within the cell wall.

Plaza (2019) reviewed recent experimental assessment of the molecular-scale interactions between wood and water, including infrared spectroscopy methods, neutron scattering and nuclear magnetic relaxometry experiments. Much of the experiments have used extracted or derived polymers. Since at molecular level, polymers might not be comparable to the in situ wood polymers in native wood, Plaza (2019) states that ‘More experimental studies that probe the unmodified wood as a whole are still needed’.

### Cell wall saturation (CWS)

Green wood contains water-saturated cell walls and lumens which can be filled to different extents with liquid water, water vapour or both. During drying, cell lumens release liquid water, and cell walls arrive in the transition between a saturated and an unsaturated state, i.e. the so-called ‘fibre saturation point (*FSP*)’ or ‘fibre saturation state’. The term as such is somewhat misleading since fibres are not saturated with water, but the cell wall is. ‘Cell wall saturation (*CWS*)’ would therefore better describe the phenomena attributed with this particular state. Such a state can be reached only theoretically. More likely, adsorption and desorption are temporarily and spatially ongoing processes which never attain to an equilibrium. However, among the first, Tiemann (1906) defined the *FSP* as the moisture content (*MC*) when lumens are empty of liquid water, cell walls begin to dry and strength begins to decrease. As previously stated by Engelund et al. (2013), this definition is problematic, since the three criteria are not fulfilled at the same *MC* (e.g. Stamm 1971), and fibre saturation is not a steady state (e.g. Hernández and Bizoň 2007).

The *FSP* can also be defined through the climatic conditions needed to achieve complete saturation of the cell walls, which should happen in equilibrium with air at 100% *RH*. Experimentally, this is hardly ever reached since minimal deviations in temperature can lead either to condensation or a drop in *RH* (Fredriksson 2019). Therefore, Popper and Niemz (2009) used the Hailwood-Horrobin model (Hailwood and Horrobin 1946) for computing *FSP* values of more than 30 different wood species to avoid conditioning of wood samples at 100% *RH*. Hoffmeyer et al. (2011) suggested an *EMC* at a matric water potential of − 0.1 MPa corresponding to 99.93% *RH*.

Wood is occasionally stored above deionized water at 20 °C to achieve full saturation of the cell walls corresponding to what is often named *FSP* (e.g. Meyer and Brischke 2015; Meyer et al. 2015b; Brischke et al. 2017, 2018a). As reported by Hunter (1995) and Fredriksson (2019), this might be incorrect since very small changes in temperature would either lower the *RH* or induce condensation, where the latter likely occurs not inside conditioned wood samples, but at the outer boundary of the conditioning room. Nevertheless, the *EMC* of seven different European-grown wood species was between 23 and 39% when stored above deionized water until constant weight in a study reported by Meyer and Brischke (2015). This coincides with early findings by Zeller (1920) who reported about 21 and 36% *EMC* above deionized water at 25 °C. Such findings support the theory of Fredriksson and Thybring (2019) that cell wall saturation (syn. fibre saturation) occurs at *RH* levels as high as those where capillary water is present in adjacent cell lumens. In other words, it is suggested that conditioning wood above deionized water does not lead to full cell wall saturation, while at the same time, capillary water is already present, and therefore the wood *MC* can be in a range between approximately 20 and 40%.

Based on Engelund et al. (2013) will a *FSP* definition based on changes in strength properties (*FSP* around 30%) or a definition based on ‘the amount of water contained within the saturated cell wall’ (*FSP* around 40%) result in a difference of about 10%. The question is why the last 10% *MC* do not affect the physical properties of the cell wall as much as the first 30% *MC*. The explanation provided was that below 30% *MC* new water molecules break different H-bonds in the wood cell polymer, while from about 30% to 40%, new water molecules are incorporated without breaking any cell wall polymer H-bonds. Regardless of how *FSP* is defined, the *FSP* can also vary based on the method used. More details about experimental techniques for characterizing water in wood covering the range from dry to fully water-saturated is found in the review by Thybring et al. (2017).

Fredriksson (2019) claimed that other techniques than those commonly used in the hygroscopic range are needed to achieve *RH* higher than 95–97% and suggested, for instance, the pressure plate technique, the pressure membrane technique, centrifuge techniques or hanging water columns. The *MC* of wood in the over-hygroscopic range (i.e. above 95% *RH*) was well correlated with the water potential and was up to 200% as reported by Cloutier and Fortin (1991), Tremblay et al. (1996) and Almeida and Hernández (2007). Also, Hunter (1995) reported about wood *MC* well above 100% between fibre saturation at 99.9% and 100% *RH*.

Fredriksson and Thybring (2019) used a novel combination of experimental techniques (i.e. pressure plate and differential scanning calorimetry) to separate total sorption hysteresis into hysteresis in cell wall water and capillary water, respectively, in the whole moisture range. They found that ‘sorption hysteresis in wood cell walls exists in the whole moisture range. The cell walls were not saturated with water until the whole wood specimen was saturated which contradicts the long-held dogma that cell walls are saturated before significant amounts of capillary water are present in wood’. Consequently, *CWS* might be considered as a quasi-stationary state, since drying and moistening of wood are processes going on in parallel. Wood conditioned at such high *RH* provides plenty of condensation nucleoli, which explains that condensation can happen significantly below *CWS*. Seemingly, the water vapour pressure gradients are barely high enough to stimulate condensation to an extent that corresponds with a wood *MC* of 100% and higher as suggested by Fredriksson (2019) who referred to estimates based on the pore structure of Norway spruce (Fredriksson and Johannsson 2016).

The definitions in literature of maximum amount of water in the wood cell wall are manifold and partly contradictory. The authors consider *CWS* as a state of wood when cell walls are completely saturated with water, i.e. cell wall water, and the cell walls are swollen at their maximum. When this happens, pores already start to get filled with capillary water. Consequently, the maximum amount of cell wall water in wood is not reached in the absence of capillary water. The latter is important for interpreting data from experiments regarding the physiological needs of decay fungi.

### Sorption isotherms

Sorption experiments have been frequently conducted in conditioning chambers with a constant climate defined by temperature (*T*) and *RH* of the air. In conventional conditioning chambers both, *T* and *RH* are subject to oscillation which is a limitation of the method (Thybring et al. 2019). More stable conditions can be achieved when exposing wood samples above different saturated salt solutions (e.g. Ammer 1963; Peralta 2007; Saito et al. 2012; Brischke et al. 2017), salt solutions of different concentration (Bavendamm and Reichelt 1938; Theden 1941) or sulphuric acid at different concentrations (Zeller 1920) at a constant temperature. Alternatively, vacuum balances of various kinds were used for determining sorption isotherms, and today, automated continuous-flow sorption balances are frequently used for measuring sorption isotherms and for studying sorption kinetics (Thybring et al. 2019). The latter technique is also known as ‘dynamic vapour sorption (DVS)’, but limited to very small samples with a mass in the milligramme range.

Fredriksson and co-workers highlighted the importance of the so-called super-hygroscopic or over-hygroscopic range (Fredriksson and Johansson 2016; Fredriksson and Thybring 2018; Fredriksson 2019) where wood takes up a substantial amount of water in a narrow *RH* range due to uptake by capillary condensation in the macro-voids, i.e. cell lumens and pit chambers. In contrast, in the hygroscopic range (i.e. between 0 and about 30% *MC*), wood absorbs water molecules in cell walls, which interact with hydroxyl groups and is bound by hydrogen bonds (Fredriksson 2019). The over-hygroscopic moisture range is the moisture range exceeding 95–98% *RH* and is sometimes also called ‘capillary moisture range’ (e.g. Nilsson et al. 2018).

Murr and Lackner (2018) found that grain size and grain layer thickness influenced the initial sorption kinetics, with the latter showing a larger impact. This confirmed the notion of a transport-limited initial mass increase, possibly due to water vapour diffusion to the sorption sites. Long-term behaviour was less affected and was attributed to the ‘concept of a relaxation and reorganisation dominated long-time behaviour’; Murr (2019) confirmed that water vapour transport influenced the sorption kinetics of small sample sizes and concluded that this result need to be considered in modelling and interpretation of water vapour sorption experiments.

Similar to other porous materials, wood exhibits sorption hysteresis. At a given climate, the *EMC* of wood is not necessarily the same, since it depends on the moisture history (Fredriksson and Thybring 2018, 2019). Usually, the *MC* is higher during desorption compared to absorption, where the amplitude of the hysteresis strongly depends on the respective ambient climate. For interpretation of experimental data on the physiological needs of decay fungi, it is therefore essential to know the moisture history of the samples and to assure that theoretical *EMC* values are either based on absorption or desorption but never on different moistening or drying regimes. Hysteresis is more pronounced at high *RH* (Fredriksson and Thybring 2019) and therefore particularly important for the interpretation of *MMThr* values. However, in most studies, specimens were used to determine *MMThr* values, which underwent absorption.

## Accessibility of sorption sites and localization of water

Hydroxyl groups (OH groups) are the predominant sorption sites for water molecules in wood. The amount of OH groups in hemicelluloses is twice as high as in lignin and four times higher compared to cellulose fibrils (Thybring et al. 2017). One approach to determine accessible OH groups in wood (in the hygroscopic range) is gravimetrically by hydrogen-deuterium exchange (Morrison 1960; Sepall and Mason 1961). The concept is initial drying, deuterium oxide (D_2_O) conditioning and final drying; deuterium oxide causes hydrogen on accessible OH groups to be exchanged with deuterium (heavy water), and the number of exchanged OH groups can be determined from change in mass. Beck et al. (2018a) reported that the OH accessibility (measured by deuterium exchange) in *Pinus radiata* earlywood stayed almost constant (6.5–8 mmol/g) during decay up to 50% *ML* caused by *Rhodonia placenta*. This might seem illogical since hemicellulose is degraded first by brown-rot fungi. It was hypothesized that new OH groups were exposed by (1) the opening of the cellulose micro-fibrils and (2) the modification of lignin by hydroxyl radicals from Fenton chemistry.

Numerous insights on wood-water relationships and fungal decay potential were derived from studies on thermally and chemically modified wood. In this regard, it has been shown that water was excluded from acetylated wood cell walls due to both direct substitution of OH groups leading to less primary sorption sites for water molecules and steric hindrance of unmodified OH groups by the bulky acetyl groups (Papadopoulos and Hill 2003; Popescu et al. 2014; Beck et al. 2017). In acetylated wood with an average weight percent gain (WPG) of 21.4%, Beck et al. (2018a) found that onset of substantial ML was preceded by a deacetylation phase. OH accessibility before decay was lower than for unmodified wood. Initiation of decay tended to increase the OH accessibility, and the reason is most likely due to oxidative degradation and deacetylation. At later stages of decay, the OH accessibility decreased again, probably due to residual acetyl groups on lignin. During the decay process, the acetylated samples never reached the OH levels found in unmodified wood.

Before and after brown-rot decay (*R. placenta*), wood-water relations were determined with low-field nuclear magnetic resonance (LFNMR) relaxometry. LFNMR can provide insights into wood-water chemical interactions as well as information about the distribution of water within the macro-void structure of the wood anatomy in the over-hygroscopic range. In acetylated wood decayed by brown rot, the behaviour of the cell wall wood-water relations (i.e. LFNMR T2 relaxation of water populations) corresponded well with the deacetylation observed by chemical characterization. Acetylation causes the water to become more mobile due to its reduced affinity for the acetylated cell wall, but the total amount of water within the cell wall is reduced (Beck et al. 2018b). Like acetylation, furfurylation was shown to reduce the amount of water within the cell wall determined with LFNMR (Thygesen and Elder 2009) However, in contrast to acetylation, furfurylation did not change the interaction of water with the cell wall surface. In the over-hygroscopic region furfurylated wood took up more water than untreated wood (voids and cracks during treatment). The same *Pinus radiata* material and test design as in Beck et al. (2017, 2018a, b) was used for furfurylated wood (Beck et al. 2019). OH accessibility in sound, furfurylated samples did not change with increasing WPG, suggesting little cross-linking occurs between the furfuryl polymer and the wood cell wall. OH accessibility in furfurylated wood samples at 32.1% WPG increased significantly after initiation of decay. This increase was attributed to opening of crystalline cellulose regions and formation of new OH groups in lignin and the furfural polymer due to oxidative alterations. The OH accessibility in sound furfurylated wood was lower than in unmodified wood, but after initiation of decay, it was slightly higher than in unmodified decayed wood (around 9 mmol/g).

Attempts to visualize capillary water in wood were made by Li et al. (2013) who used X-ray computed tomography and monitored water uptake processes in solid wood and different wood-based products. Similarly, De Ligne et al. (2019) observed density changes of small Scots pine sapwood blocks during decay by *C. puteana* with the help of X-ray CT. They hypothesized that different processes such as moisture uptake, moisture production by the fungus and *ML* due to fungal degradation caused density changes but struggled to ‘untangle these factors’. In many other studies (Watanabe et al. 2012; Lindgren et al. 2016; Hall 2019) micro-CT scanners were used to visualize and quantify wood moisture as well as its spatial distribution, for instance, during drying. However, to the best knowledge of the authors, micro-CT techniques have not yet been successfully applied to distinguish between bound and capillary water in wood during sorption or decay processes on cell and cell wall level.

## Transport processes in wood in the absence of capillary water

Fungal decay alters the sorption and electrical conductivity of wood, and an increase of accessible OH groups at initiation of decay is suggested to be linked to a change of electrical conductivity. More OH groups could contribute to a percolating network. The analysis by Zelinka et al. (2008) ‘indicates that electrical conduction in wood can be explained by percolation theory and that there exists a continuous path of Type II water in wood at w_c_, which is below the traditional fiber saturation point’. Thybring et al. (2017) suggested that an ion transport in wood is linked with the formation of a continuous network of cell wall water, and limiting cell wall moisture, e.g. chemical modification, ‘might prevent the formation of such a network, hereby disrupting the physical pathways for transport of solutes’. Jakes et al. (2013) observed that the onset of metal corrosion and fungal decay in wood occurred before capillary water is formed in cavities and aqueous chemical transport would be possible. The percolation threshold when hemicelluloses undergo glass transition is likely far below the traditional *FSP*, i.e. around 16% *MC* according to Zelinka et al. (2008) and Jakes et al. (2019).

Brischke et al. (2018b) found that brown rot and white rot reduced the sorption of wood and lowered its electrical resistance in the hygroscopic range. Decayed specimens showed a *MC* well above fibre saturation and an increased electrical resistance compared to undecayed wood at a given *MC* as long as the fungal mycelium penetrating the wood blocks was alive. The hyphae network itself served apparently as an additional pathway for ions and water. When brown-rot decayed specimens were dried and re-wetted, they showed an elevated electrical resistance beyond cell wall saturation. In white-rot decayed specimens, the resistance was reduced at a given *MC*.

Oven-drying of the specimens led to a breakdown of the gelatinous extracellular matrix (ECM) formed by the fungus (Kirker et al. 2017). Afterwards, changes in electrical conductivity became apparent but were the consequence of the respective degradation patterns of brown- and white-rot fungi. During active fungal infestation, the fungi-induced changes of the cell wall chemistry are overruled by the presence of liquid water, not only in cell wall voids but also in the cell lumens.

Jakes et al. (2019) reviewed and applied approaches established in polymer science as a tool to understanding the effects of moisture on diffusion in unmodified wood cell walls. The premise was that ‘the movement of chemicals through wood cell walls is a diffusion process through a solid polymer’ and in contrast to previous assumptions of aqueous pathways. They conclude that both lignin and the amorphous polysaccharides in wood are likely to have glass transitions. Glass transition temperatures are affected by moisture and will increase when moisture decrease. This is of importance because diffusion strongly depends on the state of the polymer (i.e. rigid glassy state or soft rubbery state). The effects of water sorption and plasticization are not directly proportional, and water in ‘holes’ does not contribute to plasticization. The implications regarding fungal decay are not explicitly mentioned by Jakes et al. (2019), but several of their findings can be important to fungal metabolism, for example: (1) the parallel existence (in time and space) of different proposed states of water could help explain why fungal decay sometimes seems to start at wood *MC* below the traditional *FSP*, and (2) absorption of water molecules in ‘holes’ and the formation of water clusters might serve as ‘initiation spots’ of fungal enzymatic activity (provided the enzymes have access) and ‘may provide avenues for aqueous diffusion of chemicals through cell walls’. Crucial questions that arise are: (1) Is wood a miscible blend, a compatible blend, or an immiscible blend? (i.e. what is the glass transition point of wood cell walls?), and (2) does fungal decay depend on the glass transition point of a single component? (i.e. do fungi start to degrade hemicelluloses as soon as their enzymes can diffuse into them?). They further highlight a difference in diffusion through wood polymers vs. typical polymers, the high swelling pressures that can develop in unmodified wood cell walls and that this pressure should be given attention in future diffusion models.

## Quantification of fungal responses

As an alternative to traditional mass and strength loss measurements, the metabolic activity of wood-decay fungi can be determined by microcalorimetry where the heat production rate is measured (Xie et al. 1997; Bjurman and Wadsö 2000; Wadsö et al. 2013; 2017). Bjurman and Wadsö (2000) applied the technique for studying the effect of temperature on fungal decay. Wadsö et al. (2013) studied the effect of different *MC* on fungal metabolism, and Wadsö et al. (2017) aimed on utilizing isothermal microcalorimetry for determining the durability of different wooden materials against fungal decay. Calorimetry measurements are very sensitive to small changes in fungal metabolic activity, and measurements can be conducted continuously. Hence, they are outperforming *ML* measurements for monitoring fungal decay development. One limitation of this method, and most other methods with a very well-controlled environment, is the limited number of samples allowed for each experimental run, often only one.

DNA-based methods are powerful tools for identification of wood-decaying organisms and for quantification of fungal biomass. Profiling of fungal communities related to wood protection include different materials exposed in experimental test fields (Råberg et al. 2007, 2009, 2013; Prewitt et al. 2014) and fence poles (Råberg and Daniel 2009). Profiling of species succession during decay of different wood materials using molecular tools is very limited. Råberg et al. (2007) compared species composition in six German test fields for two preceding years using terminal restriction fragment length polymorphism (T-RFLP), cloning and subsequent sequencing (semi-destructive sampling by drilling). Jacobs et al. (2019) studied fungal community succession in pine and beech stakes every half year, over a period of 3 years, using both morphological methods and DNA analysis (destructive sampling). None of the studies have taken moisture into account. The challenge of identification of fungal communities in field samples is that the analysis requires only a small amount of sample. To represent even a relatively small field test stake, a high number of replicates must be taken to get an estimate of the fungal community. And the fungal community will change over time. Reproducibility is challenging even within the same test site, a larger variation will occur between sites because of differences in inoculum potential, temperature and moisture. Hence, from an applied aspect, it does not so much focus on which fungal species decay the wood but on the resistance of the material against fungal degradation in general.

Methods to quantify fungal biomass in wood samples include ergosterol or chitin assays (traditional biomass assays, e.g. Matcham et al. 1985; Schnürer 1993) or DNA quantification with quantitative real-time PCR (qRT-PCR) (Eikenes et al. 2005). qRT-PCR has the advantage that it can be used for identification and quantification on species level (species specific primer) or at group level (e.g. basidiomycete-specific primer). When compared to chitin and ergosterol, qRT-PCR was shown to be the most sensitive method both in laboratory (Eikenes et al. 2005) and for field test stakes (Pilgård et al. 2011).

Unlike the genome, which is roughly fixed, the transcriptome can vary with external environmental conditions. The transcriptome reflects the genes that are being actively expressed at any given time. Gene expression studies of untreated wood have provided new insight regarding basidiomycete decay mechanisms (e.g. Sato et al. 2009; Martinez et al. 2009; MacDonald et al. 2011, 2012; Van den Wymelenberg 2009, 2010, 2011; Suzuki et al. 2012; Doria et al. 2014; Gaskell et al. 2014; Zhang et al. 2016; Zhang and Schilling 2017). Gene expression studies on preservative-treated (e.g. Kang et al. 2009a, b; Tang et al. 2013) and preservative-modified wood are still relatively limited (Alfredsen and Pilgård 2014; Ringman et al. 2014, 2015; Alfredsen et al. 2016a, b; Beck et al. 2018b; Skrede et al. 2019; Kölle et al. 2019). To the best knowledge of the authors, no experiments have been done on gene expression where the focus was to study the effect of different moisture levels. One reason is that it would be technically challenging to keep the wood moisture stable throughout the decay test. But the effect of moisture on fungal gene expression should, indirectly, have been captured in the modified wood experiments. Wood modifications have been claimed to have a non-toxic mode of action against decay fungi, and the lower *EMC* is believed to be the main effect against decay fungi as recently reviewed by Ringman et al. (2019). According to recent gene expression studies (Beck et al. 2018a; b; Skrede et al. 2019), the fungus starts a common decay process in the modified wood but proceeds at a slower pace. The slower process in modified wood could be due to reduced access to cell wall polysaccharides and/or lower *EMC*. The lower *EMC* will result in reduced transport of enzymes or water in an inadequate location or form. Ringman et al. (2019) review in more detail the role of water in brown-rot decay of chemically and thermally modified wood.

Important aspects that should be given more attention in future gene expression studies of wood-decomposing fungi are: (1) specimen design and harvest intervals (Zhang et al. 2016; Kölle et al. 2019), (2) substrate/culture conditions (Wu et al. 2018; 2019), (3) relevant comparison between treatments, (4) how to handle reference genes for accurate normalization (Zhang et al. 2019b) and (5) in situ mRNA hybridization rather than bulk sampling (Zhang et al. 2019a). It is worth to keep in mind that according to Vogel and Marcotte (2012) ∼ 60% of variation in protein concentration cannot be explained by measuring mRNAs alone. Hence, there is a need to expand the knowledge of what is secreted, especially for brown-rot fungi, and using a realistic substrate, i.e. solid wood (Presley and Schilling 2017; Presley et al. 2018; Wu et al. 2018).

## Conclusions

Research on both wood-water relationships and the physiological needs of wood-decaying fungi has been consecutively performed during the last 150 years. Regardless, the interrelationships between moisture dynamics in wood and its effect on the activity of decay fungi are still not fully understood. Fortunately, respective research activities have been intensified during recent years. From the review of these rather complex interactions, one might conclude the following:

- Understanding the moisture requirements of decay fungi is key for interpreting wood durability test data, for analysing the protective mode of action of new wood protection systems and for accurate modelling of degradation processes and the resulting service lifetimes of wood products.
- Because of the assumed non-toxic effect and the change in *MC*, studies on fungal decay of modified wood provide insight regarding the wood-water effect on fungal behaviour. In the future, more targeted modifications (e.g. Digatis et al. 2019) of the cell wall, to increase or decrease moisture, could provide important new insight.
- Quantification and localization of capillary and cell wall water – especially in the over-hygroscopic range – is considered crucial for determining minimum moisture thresholds (*MMThr*) of wood-decay fungi. In particular, the role of capillary or loosely bound water in modified wood necessitates clarification, i.e. it is still not understood whether decay fungi can utilize capillary water in cell lumens or larger cell wall voids for metabolizing cell wall substance. Increased knowledge about the potential transport processes in wood in the absence of capillary water might add additional pieces to this puzzle.
- Further unknowns are the minimum wood volume that needs to exceed a certain *MMThr* and the time needed to allow for onset of decay under such marginal conditions. In this respect and for practical purposes, it is also interesting to increase the understanding about the effect of dry periods on fungal mycelium and its ability to get revitalized after re-wetting.
- The limitations of the various methods and experimental set-ups to investigate wood-water relationships and their role for fungal decay are manifold. Hence, combining techniques from wood science, mycology, biotechnology, and advanced analytics, such as calorimetry, DVS, DSC, LFNMR, fungal transcriptome and secretome, microspectroscopy and chemometrics with sub-cell wall spatial resolution using incubation experiments on solid wood substrate in strictly controlled environments might provide new insights and eventually a breakthrough in understanding.
- It is commonly agreed that knowledge about how fungi sense the dynamic composition of the wood cell wall (incl. water amount and distribution) and adapt their secretome in response are still fragmentary. Still, huge efforts are needed to close the existing gaps in our understanding of fungal biodegradation. The detailed laboratory studies suggested might seem irrelevant from an applied perspective. But a breakthrough on a detailed level will potentially have big direct implications for different applications. Examples include, e.g. targeted blocking of fungal metabolic pathways for improved wood protection, precision of modelling tools and new enzymes or enzyme combinations for biorefinery applications. Regarding the bigger picture, climate change is the grand challenge of our time. Since more than half of all biomass on Earth is wood, part of the solution is improved utilization and longer service life (i.e. carbon storage) and valorization (i.e. biorefinery applications) of the renewable wood source as a substitute for more energy-consuming raw material sources. By unravelling the intricate details about decomposition of wood by lignocellulolytic fungi, we will also have tools to better quantify carbon storage and release both in nature and for wood in service.
