# Correction to: Wood-water relationships and their role for wood susceptibility to fungal decay

**DOI:** 10.1007/s00253-021-11408-6

**Published:** 2021-08-02

**Authors:** Christian Brischke, Gry Alfredsen

**Affiliations:** 1 Department ofWood Biology and Wood Products, Faculty of Forest, Sciences and Forest Ecologylm, University of Goettingen, Buesgenweg, 4, D-37077 Goettingen, Germany; 2 Division of Forest and Forest Resources, Wood Technology, Norwegian Institute of Bioeconomy Research (NIBIO), Høgskoleveien 8, 1433 Ås, Norway

**Correction to: Applied Microbiology and Biotechnology (2020) 104:3781–3795**

10.1007/s00253-020-10479-1

The article “Wood-water relationships and their role for wood susceptibility to fungal decay,” written by Christian Brischke and Gry Alfredsen, was originally published Online First without open access. After publication in volume 104, issue 9, pages 3781–3795, the author decided to opt for Open Choice and to make the article an open access publication. Therefore, the copyright of the article has been changed to © The Author(s) 2020 and the article is forthwith distributed under the terms of the Creative Commons Attribution 4.0 International License (http://creativecommons.org/licenses/by/4.0/), which permits use, duplication, adaptation, distribution, and reproduction in any medium or format, as long as you give appropriate credit to the original author(s) and the source, provide a link to the Creative Commons license, and indicate if changes were made. The images or other third party material in this article are included in the article’s Creative Commons licence, unless indicated otherwise in a credit line to the material. If material is not included in the article’s Creative Commons licence and your intended use is not permitted by statutory regulation or exceeds the permitted use, you will need to obtain permission directly from the copyright holder. To view a copy of this licence, visit http://creativecommons.org/licenses/by/4.0. Open access funding enabled and organized by Projekt DEAL.

The original article was corrected.

